# Ferroptosis as a Novel Therapeutic Target for Diabetes and Its Complications

**DOI:** 10.3389/fendo.2022.853822

**Published:** 2022-03-29

**Authors:** Xi-Ding Yang, Yong-Yu Yang

**Affiliations:** ^1^Department of Pharmacy, The Second Xiangya Hospital of Central South University, Changsha, China; ^2^Phase I Clinical Trial Center, The Second Xiangya Hospital of Central South University, Changsha, China; ^3^Hunan Provincial Engineering Research Central of Translational Medical and Innovative Drug, The Second Xiangya Hospital of Central South University, Changsha, China

**Keywords:** ferroptosis, diabetes, diabetic complications, diabetic kidney disease, iron

## Abstract

The global diabetes epidemic and its complications are increasing, thereby posing a major threat to public health. A comprehensive understanding of diabetes mellitus (DM) and its complications is necessary for the development of effective treatments. Ferroptosis is a newly identified form of programmed cell death caused by the production of reactive oxygen species and an imbalance in iron homeostasis. Increasing evidence suggests that ferroptosis plays a pivotal role in the pathogenesis of diabetes and diabetes-related complications. In this review, we summarize the potential impact and regulatory mechanisms of ferroptosis on diabetes and its complications, as well as inhibitors of ferroptosis in diabetes and diabetic complications. Therefore, understanding the regulatory mechanisms of ferroptosis and developing drugs or agents that target ferroptosis may provide new treatment strategies for patients with diabetes.

## Introduction

According to the International Diabetes Federation, 536.6 million adults are affected by diabetes worldwide (2021), and the total number of adults with diabetes is predicted to rise to 783.2 million by 2045 (1). The World Health Organization proposes that diabetes will be the seventh leading cause of death by 2030 (2). There are two major forms of diabetes: type 1 and type 2 diabetes mellitus (T2DM). Type 1 diabetes mellitus (T1DM) is caused by insufficient insulin secretion, and T2DM results from insulin resistance (3). Intervention studies have confirmed that hyperglycemia is a major cause of diabetic complications (4). Long-term hyperglycemia damages multiple organs, including the kidneys, cardiovascular system, nerves, bones, and eyes, ultimately resulting in severe complications (5). Major diabetic complications include diabetic kidney disease (DKD), retinopathy (DR), neuropathy (DNP), osteoporosis (DOP), and cardiomyopathy (DCM). With increasing trends in diabetes worldwide, mortality and associated costs owing to diabetes and diabetes-related complications are a major global public health concern (6). It is generally believed that intensifying glucose control reduces the risk of diabetic complications and ameliorates them (7). However, the occurrence and progression of diabetic complications cannot be prevented in some patients (8).

As diabetes progresses, persistent hyperglycemia increases the levels of inflammatory factors, advanced glycation end products (AGEs), reactive oxygen species (ROS), free fatty acids (FFAs), triacylglycerol, and diacylglycerol in the heart, retina, kidneys, and nervous system (9–12). These factors individually and/or synergistically result in cellular death, including apoptosis, autophagy, and necroptosis (9, 10, 13). Diabetes complications are believed to be a consequence of cellular death (14–16). For example, increasing evidence demonstrates that death of pancreatic B cells is the main cause of insufficient insulin secretion in diabetes (17). Similarly, cell death is associated with diabetes complications such as DKD (14), DR (18), DN (19), DCM (15), and DOP (20).

Ferroptosis is a recently recognized form of programmed cell death that is genetically, biochemically, and morphologically distinct from apoptosis, necroptosis, autophagy, and other types of cell death (21, 22). In recent years, ferroptosis has been found to be involved in diabetes and its multiple complications (23–32). Thus, pharmacological modulation of ferroptosis has emerged as a promising therapeutic strategy for diabetes and various diabetic complications. In this study, we provide an update on the contribution of ferroptosis to the pathogenesis of diabetes and its complications. Potential therapeutic drugs or compounds targeting the ferroptosis pathway for diabetic complications are also discussed.

## Overview of Ferroptosis

In 2003, a new compound, erastin, was found to induce a new way of cell death (33). This mechanism of cell death can be inhibited by iron-chelating agents (34). In 2012, Dixon first named this cell death pattern as ferroptosis (35). Ferroptosis differs from apoptosis, necroptosis, and autophagy in terms of cell morphological characteristics and functions (21, 22). Ferroptosis does not exhibit the typical morphological characteristics of necrosis, such as swelling of the cytoplasm and organelles and rupture of the cell membrane (36). It also does not result in morphological changes similar to typical apoptosis, including cell shrinkage, chromatin condensation, and the formation of apoptotic bodies (37). Ferroptosis does not form a classical closed bilayer membrane structure, which is the main difference between ferroptosis and autophagy (38).

**Figure 1:**
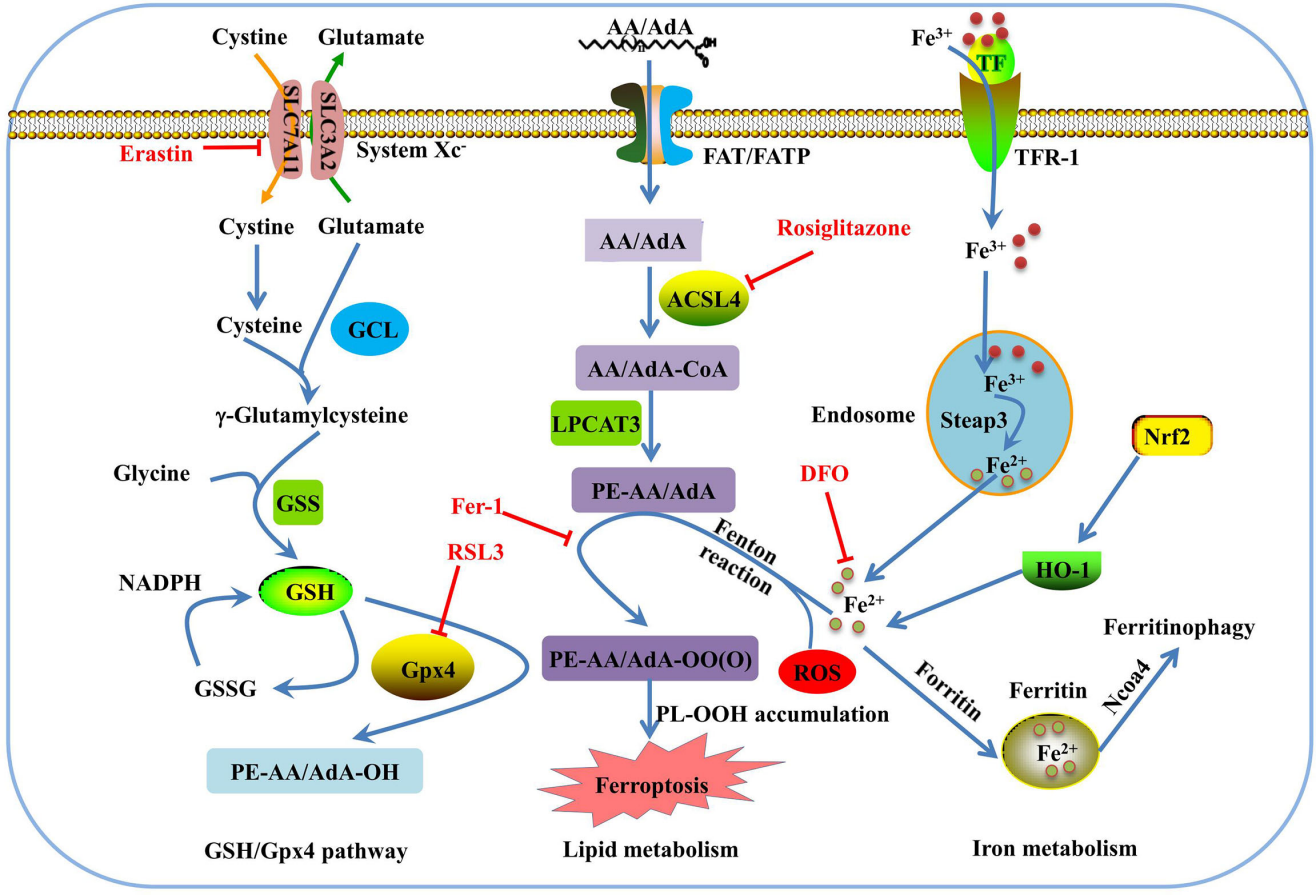
Overview of the ferroptosis pathway. GSH is synthesized from glutamate, cysteine, and glycine, and catalyzed by glutamate cysteine ligase (GCL) and glutathione synthetase (GSS). GPX4 converts GSH to glutathione oxidized (GSSG) to inhibit lipid ROS production. Erasin and RSL3 trigger ferroptosis by inhibiting of system Xc- and Gpx4. In lipid metabolism pathway, phosphatidylethanolamine-adrenic acid/arachidonic acid (PE-AA/AdA) is synthesized in two steps catalyzed by Acyl-CoA synthetase long-chain family member 4 (ACSL4) and lysophosphatidylcholine acyltransferase 3 (LPCAT3). Rosiglitazone is the strongest inhibitor of ACSL4 that alleviates ferroptosis. Transferrin receptor 1 (TFR-1), transferrin (TF), ferritin, and Ncoa4 are involved in iron metabolism and are closely associated with ferroptosis. Deferoxamine (DFO) and ferrostatin-1(Fer-1) inhibit ferroptosis by regulating iron level and lipid oxidation, respectively.

Morphologically, ferroptosis is mainly characterized by the shrinkage of mitochondria with increased membrane density, as well as the decrease or disappearance of mitochondrial cristae (39). Biochemically, ferroptosis is characterized by iron-dependent lipid peroxidation (35, 40). Cysteine deficiency and glutathione (GSH) synthesis inhibition contribute to ferroptosis (22, 41), while iron-chelating agents and lipophilic antioxidants can prevent ferroptosis (42). The pathways in the ferroptosis cascade can be roughly divided into three major categories: GSH/glutathione peroxidase 4 (Gpx4) pathway, iron metabolism, and lipid metabolism (**Figure 1**). Currently, the molecular mechanisms underlying ferroptosis remain elusive. To date, multiple proteins, such as voltage dependent anion channel 2/3, p53, nuclear receptor coactivator 4 (NCOA4), cysteinyl-tRNA synthetase (CARS), mitogen-activated protein kinase (MAPK), nicotinamide adenine dinucleotide phosphate oxidase (NOX), and other proteins, can positively regulate ferroptosis (43–48). In addition, some genes are involved in the negative regulation of ferroptosis, including *Gpx4*, nuclear factor erythroid 2-related factor 2 (*Nrf2*), heat shock protein beta-1(*HSPB1*), and system Xc^-^ (*xCT*), which protect cells against ferroptosis (49–52).

## Effects and the Molecular Mechanisms of Ferroptosis on Diabetes and Diabetes-Related Complications

**Figure 2:**
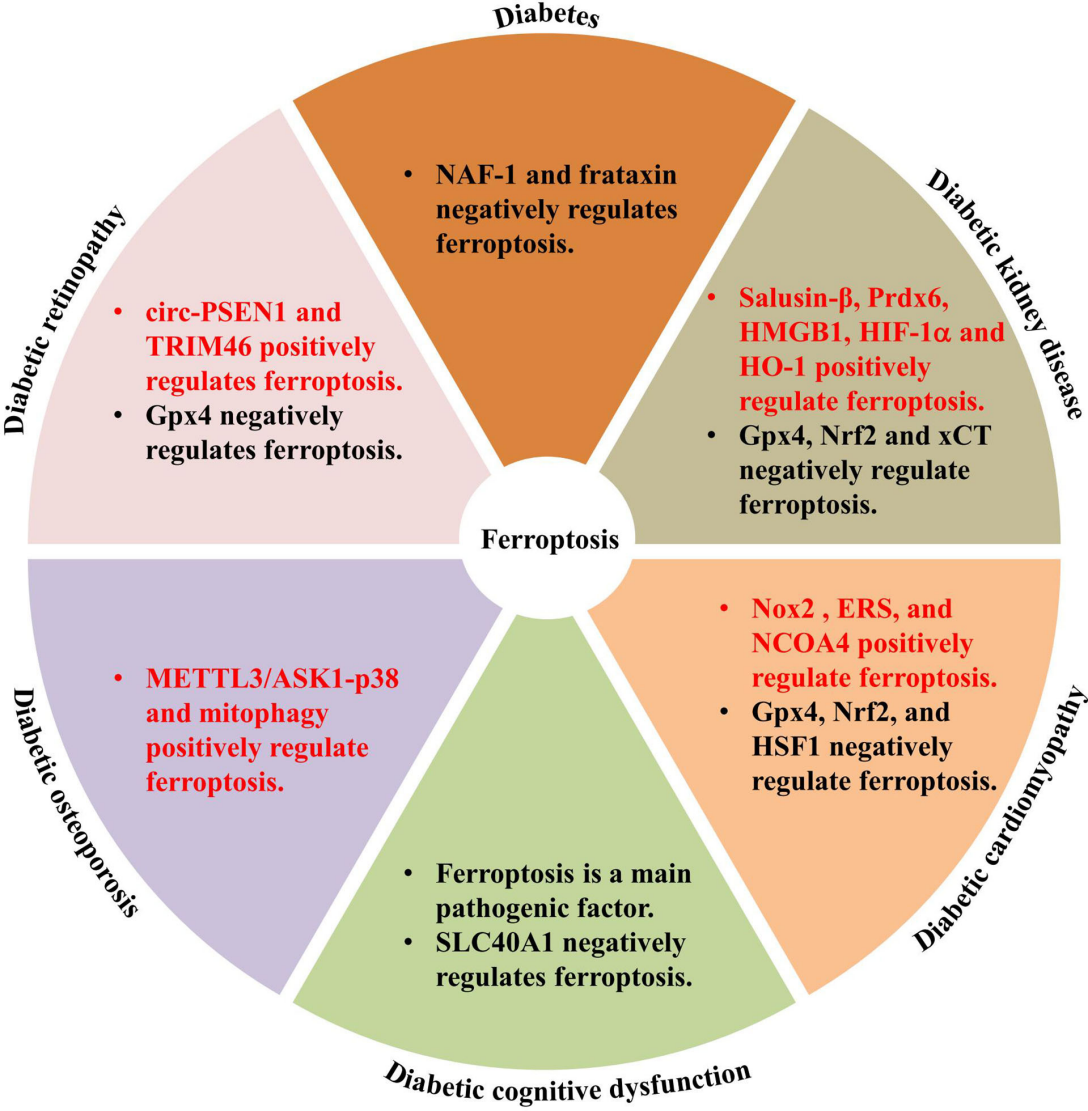
Genes involved in ferroptosis regulation in diabetes and diabetic complications.

Ferroptosis is involved in various human diseases, such as acute kidney injury (53), cancers (54–56), Parkinson’s disease (PD) (57), and other neurodegenerative diseases (58). As a new mechanism of cell death, the discovery of ferroptosis and the mechanisms involved in its regulation may help to develop novel treatments for several diseases. Recent studies have documented significant effects of ferroptosis on the development and progression of T1DM (59); T2DM (60, 61); gestational diabetes mellitus (60–62); and diabetes complications such as DKD (27), DCM (29), DR (63, 64), diabetes-induced endothelial dysfunction (32), cognitive dysfunction (31), wound healing (65), diabetic atherosclerosis (66), and DOP (67). The molecular mechanisms of ferroptosis in diabetic complications involve multiple proteins, including acyl-CoA synthetase long chain family member 4 (ACSL4), high-mobility group box-1 (HMGB1), hypoxia-inducible factor-1α (HIF-1α), heme oxygenase-1 (HO-1), tripartite motif containing 46 (TRIM46), circ-PSEN1, NCOA4 (68), and Nox2, and negative genes, *Gpx4*, *Nrf2*, *xCT*, adenosine monophosphate-activated protein kinase, heat shock factor 1 (*HSF1*), and nutrient-deprivation autophagy factor-1 (*NAF-1*) (69) (**Figure 2**). The key molecular mechanisms underlying ferroptosis in diabetes and its complications are discussed in detail below.

### Ferroptosis and Diabetes

Excessive iron stores have been demonstrated to be associated with the development of T2DM, and ferritin levels are increased in T2DM (70) and gestational diabetes mellitus patients (71). The activity of islet cells is closely associated with diabetes, and insufficient insulin secretion caused by pancreatic β-cell failure contributes to hyperglycemia. Increased cellular iron modulates the expression of genes involved in β-cell function and causes pancreatic β-cell dysfunction (72). Erastin, a small potent molecule capable of selectively inhibiting the Xc-cystine/glutamate antiporter required for GSH biosynthesis, induces ferroptosis (73) and affects the growth and function of human pancreatic islet-like cell clusters (74). NAF-1, a member of the [2Fe-2S] NEET protein family, regulates mitochondrial iron levels (75). Suppressed expression of NAF-1 in INS-1E pancreatic β-cells results in the appearance of ferroptosis-like features characterized by enhanced lipid peroxidation, decreased expression of Gpx4, and enhanced expression of tTfR. Fer-1 treatment of INS-1E NAF-1(−) cells significantly reduced ferroptosis and improved cell growth (69).

Acrolein and arsenic are common environmental pollutants that threaten public health (76, 77). Growing evidence has revealed that chronic arsenic exposure is a high-risk factor for T2DM (78, 79). Arsenic induced ferroptosis *in vitro* in a dose-dependent manner. Ferroptosis occurs in animal models of arsenic-induced pancreatic dysfunction (61). The ferroptotic effect of arsenic on mouse insulinoma cells depends on regulation of the mitochondrial ROS-autophagy-lysosomal pathway (61). In mouse pancreatic β-cell MIN6 cells, acrolein induces ferroptosis and insulin secretion dysfunction *via* the endoplasmic reticulum (ER) stress-related PERK pathway (77). These data indicate that ferroptosis plays an important role in maintaining homeostasis in the pancreatic islet cells.

In addition to their involvement in the regulation of islet cell death and dysfunction, ferroptosis-related proteins modulate diabetes-related metabolic phenotypes. Friedreich’s ataxia (FRDA) is a neurodegenerative disease caused by a defect in mitochondrial frataxin (FXN), a key regulator of ferroptosis, and patients with FRDA are predisposed to diabetes (80). FXN knock-in/knock-out (KIKO) mice exhibited hyperlipidemia, reduced energy expenditure and insulin sensitivity, and reduced thermogenic activities of brown adipose tissue. Moreover, the mRNA levels of the iron modulators (Ncoa4 and solute carrier family 39 member 14) were upregulated in brown adipocyte precursors of KIKO mice. Increased susceptibility of brown adipocyte precursors to ferroptosis could be one of the causes of brown adipose tissue (BAT) dysfunction (81). These data suggest that ferroptosis and its related proteins are involved in disorders of glucose and lipid metabolism. However, little is known about the exact mechanisms by which iron dysmetabolism affects metabolic phenotypes *in vivo*, and further studies are needed.

### Ferroptosis and DKD

DKD is a major complication of diabetes and is characterized by proteinuria and decreased glomerular filtration rate (82). Accumulating evidence has indicated that hyperglycemia-stimulated oxidative stress, AGE production, inflammation, and fibrosis are all involved in the pathogenesis of DKD (83–85). Iron homeostasis is essential for normal functioning of renal cells (86). In patients with DKD, the indicators of ferroptosis, including the release of serum ferritin and lactate dehydrogenase, are upregulated (24). In kidney biopsy samples from patients with DKD, *xCT* and *Gpx4* mRNA expression was also reduced compared to that in control samples (26). A low-iron diet or iron chelator can delay the progression of DKD in diabetic rats (87). Recent studies have demonstrated that ferroptosis is involved in the development of DKD and may be a new approach to explore the progression of DKD (23–26, 87).

Animal experiments showed that iron content was increased in the kidney tissue of streptozotocin (STZ)-induced DKD mice and diabetic (db/db) DKD mice, especially in the renal tubules. The ACSL4 inhibitor rosiglitazone improves kidney function in DKD mice and reduces the lipid peroxidation product and iron content, and these effects are correlated with attenuated ferroptosis (23). Renal tubular injury is a critical factor in DKD. High glucose (HG) triggered iron overload, antioxidant capability reduction, massive ROS production, and lipid peroxidation in renal tubular cell death (23, 88). Salusin−β, a bioactive peptide of 20 amino acids, participates in HG−induced HK−2 cell ferroptosis in an Nrf−2−dependent manner (88). Feng et al. found that ferroptosis might damage renal tubules in diabetic models *via* the HIF-1α/HO-1 pathway (25). Another study revealed that inhibition of ferroptosis by upregulating Nrf2 could delay the progression of DKD (27). Transforming growth factor-β1 (TGF-β1) is an important factor that leads to renal fibrosis, and renal tubular cell death induced by TGF-β1 is known to be involved in DKD (89). GSH concentration and xCT and Gpx4 expression were significantly decreased, and lipid peroxidation was enhanced in TGF-β1-stimulated renal tubular cells. These changes were significantly ameliorated by Fer-1 (26).

Mesangial cells, specialized smooth muscle cells, are distributed between the capillary loops of glomerular capillaries, and their injury is a basic pathological change in DN (90). High mobility group box 1 **(**HMGB1**)** has been reported to be a damage-associated molecular pattern molecule, and its levels are elevated in patients with DKD. Further results identified HMGB1 as having a regulatory role in the ferroptosis of mesangial cells. Suppression of HMGB1 restores cellular proliferation, prevents ROS and lactate dehydrogenase (LDH) generation, decreases ACSL4, and increases Gpx4 levels in mesangial cells (24). Ferroptosis is also involved in podocyte injury in diabetic conditions. Podocytes are an important component of the glomerular filtration barrier (GFB), and podocyte damage is considered one of the main mechanisms leading to GFB injury (91). An *in vitro* study demonstrated that HG triggered ferroptosis in mouse glomerular podocyte MPCs, and these effects were mediated by peroxiredoxin 6 (Prdx6), a new member of antioxidant enzymes (92). Taken together, the above studies demonstrate that ferroptosis is closely related to the pathogenesis of DKD and promotes DKD through a variety of mechanisms in podocytes, mesangial cells, and tubule cells. An in-depth study of the pathological mechanism of ferroptosis would help in the timely and reasonable prevention and treatment of DKD by targeted ferroptosis.

### Ferroptosis and DCM

DCM, characterized by diastolic and systolic dysfunction, left ventricular hypertrophy, myocyte hypertrophy, and fibrosis (93), is a major cause of heart failure in patients with diabetes. Increasing evidence suggests that ferroptosis is involved in the pathogenesis of cardiomyocyte injury (28, 29). Wang et al. first found that diabetes can cause more severe myocardial ischemia-reperfusion injury (I/RI) by promoting ferroptosis in myocardial cells, as well as two other forms of programmed cell death, including apoptosis and pyroptosis. However, it is not known which type of cell death has the dominant function in DCM (28). However, what is certain is that cardiomyocytes of diabetic myocardial I/RI rats were injured accompanied by increased ferroptosis level. Inhibition of ferroptosis by Fer can alleviate this injury. ERS aggravates hypoxia/reoxygenation (H/R)-induced cardiomyocyte injury and inhibition of ERS alleviates ferroptosis (29). Ferritinophagy is a newly identified selective autophagy pathway that is mediated by NCOA4. NCOA4 mediates ferritin degradation in the autophagosome and causes the release of iron ions from ferritin, resulting in ferroptosis (94). Knockdown of NCOA4 reduced ferritinophagy and ferroptosis in a high-glucose hypoxia reoxygenation model (68).

Excessive saturated fatty acids, such as palmitic acid (PA), resulting from lipid metabolism in patients with diabetes, have been shown to play a role in cardiomyocyte death and in the development of DCM (95). It is widely accepted that apoptosis and necroptosis contribute greatly to PA-induced myocardial injury (96–98). Ferroptosis is also known to be involved in PA-induced myocardial injury. Ferroptosis inhibitors significantly reduced cell death in H9c2 cardiomyoblasts exposed to PA (30). HSF1 is an important defender of ferroptosis in cardiomyocytes. HSF1 negatively regulates PA-induced cell death, and HSF1^-/-^ mice treated with PA exhibited more serious ferroptosis by decreasing SLC40A1, FTH1, and Gpx4 expression and increasing TFRC expression in the heart (30). Nrf2, a transcription factor controlling the expression of many ferroptosis-related genes such as Gpx4, is generally considered to have an inhibitory effect on ferroptosis (99). An interesting study demonstrated that Nrf2 has detrimental effects on the heart by promoting ferroptosis during myocardial autophagy deficiency (100). However, targeting Nrf2 and its related targets remains a viable approach to prevent or treat DCM by regulating ferroptosis. In summary, these findings suggest that ferroptosis is involved in the pathogenesis of DCM. At present, the mechanism of ferroptosis in DCM remains poorly understood, and areas related to ferroptosis and cardiomyocyte function need to be further explored.

### Ferroptosis and Diabetic Neurodegenerative Disease

Diabetes is a risk factor for neurodegenerative disorders such as Alzheimer’s disease (AD), PD, Huntington’s disease, amyotrophic lateral sclerosis, and FRDA (101). Ferroptosis is involved in the development of neurodegenerative diseases (102). In some neurodegenerative diseases, iron is redistributed in specific regions of the central and peripheral nervous systems; for example, iron content is significantly increased in the hypothalamus of patients with AD (103) and in the dopaminergic neurons of the substantia nigra in PD (104). AD is characterized by a loss of neurons in the frontal lobe, amygdala, and hippocampus. Hao et al. (31) reported that ferroptosis is the main pathogenic factor in diabetes-induced cognitive dysfunction. Among the ferroptosis signaling pathway genes, the *SLC40A1* gene (ferroportin) was significantly downregulated in the diabetic rat hippocampus (31). Hyperglycemia or hypoglycemia can cause nerve cell injury, resulting in cognitive dysfunction. Another study showed that ferroptosis occurs in the hippocampus of db/db mice, and liraglutide, a drug approved for the treatment of obesity and diabetes, reduces ferroptosis by suppressing oxidative stress and iron overload, improving hippocampal neuronal and synaptic plasticity, thereby restoring cognitive impairment (105). In diabetic rats, but not in control rats, an iron chelator, deferoxamine, improved sensorimotor and cognitive outcomes of rats subjected to thromboembolic middle cerebral artery occlusion (106). These data indicate that the inhibition of ferroptosis may prevent brain injury and provide a novel disease-modifying therapeutic strategy for the prevention of cognitive impairment in diabetes.

### Ferroptosis and DR

DR is one of the most devastating complications of diabetes, affecting millions of working-age adults worldwide (107). DR is a leading cause of global vision loss. Retinal microvasculopathy, inflammation, and retinal neurodegeneration are the major causes of DR. Hyperglycemia causes pericyte damage, endothelial cell dysfunction, and basement membrane thickening in retinal vessels, leading to disruption of the blood-retinal barrier (108). Dysfunction and increased permeability of the retinal capillary endothelial cells (RCECs) are essential features of DR progression. HG inhibits human RCEC growth and induces ferroptosis, which can be reversed by Fer-1. TRIM46 mediated Gpx4 ubiquitination pathway is involved in HG-induced ferroptosis in human RCECs (63). In addition to microvascular changes, retinal neurodegeneration or loss of the retinal pigment epithelium (RPE) also contribute to diabetic retinal damage early in DR, and HG has detrimental effects on RPE cells by producing ROS and decreasing the Gpxs expression (109). An *in vitro* study in ARPE-19 cells showed that circular RNAs (circRNAs) play a regulatory role in HG-induced ferroptosis *via* competing endogenous RNAs to regulate target microRNAs (miRNAs). Circ-PSNE1 was significantly upregulated in DR patients and in HG-treated ARPE-19 cells, and downregulation of circ-PSEN1 reduced cell death, decreased MDA and Fe^2+^ content, and increased the expression of Gpx4 and solute carrier family 7 member 11 (SLC7A11). Furthermore, Zhu, et al. demonstrated that circ-PSEN1 mitigates HG-induced ferroptosis *via* the miR-200b-3p/cofilin-2 axis (64). These studies suggest that ferroptosis plays an important role in damage to RCECs and retinal pigment epithelium under hyperglycemic conditions. However, whether ferroptosis is involved in HG-triggered pericyte loss remains unclear and requires further investigation.

### Ferroptosis and DOP

Recently, osteoporosis has been recognized as a complication of diabetes, and DOP has a serious impact on the quality of life of the elderly. A decrease in bone mineral density (BMD) is a characteristic of patients with T1DM. Conversely, most studies have demonstrated that BMD does not decrease in patients with T2DM (110). Although seemingly paradoxical, the fracture risks in patients with T1DM and T2DM are similar (111). Although BMD is variable in T1DM and T2DM, prolonged hyperglycemia promotes changes in bone metabolism and destroys bone micro-architecture through multiple mechanisms, such as production of AGEs, inflammation, calcium and phosphorus metabolism disorders, and oxidative stress (112). DOP has also been reported to be associated with ferroptosis, and iron overload can promote osteoclast differentiation and bone resorption through ROS production (113). HG induces ferroptosis, and Fer-1 inhibits the downregulation of the expression of osteogenesis-associated proteins, osteoprotegerin (OPG) and osteocalcin (OCN), in MC3T3-E1 cells, suggesting that ferroptosis is involved in osteogenic ability in the presence of HG (20). Recent studies have shown that Fer-1 and DFO block ferroptosis induced by HG and palmitic acid (HGPA) in MC3T3-E1 cells. The mechanism of high glucose and high fat (HGHF)-induced ferroptosis in osteoblasts is related to the activation of the METTL3/ASK1-p38 signaling pathway (114). Morphological changes of mitochondrial are the important hallmark of ferroptosis, and studies have shown that mitochondria play an important role in the regulation of ferroptosis *via* regulation of energetic metabolism, iron metabolism, and other pathways (115, 116). Mitochondrial ferritin (FtMt), an iron-storage protein, can intercept excessive iron ions to decrease ROS levels in mice and is involved in osteoblastic ferroptosis in type 2 DOP. Overexpression of FtMt reduced osteoblastic ferroptosis and improved osteoblast function under HG conditions, whereas knockdown of FtMt decreased osteogenic function and induced mitophagy through the ROS/PINK1/Parkin pathway (67). These studies indicate that the occurrence of T2DOP is correlated with iron homeostasis imbalance and ferroptosis in osteoblasts. However, the detailed mechanism requires further investigation.

## Ferroptosis Inhibitors in Diabetes and Diabetic Complications

With an in-depth study on the mechanism of ferroptosis, many specific inhibitors of ferroptosis have been identified, such as Fer-1, deferoxamine, liproxstatin-1, mitoquinone, vitamin E, and zileuton (38, 49). Ferroptosis inhibitors in diabetes and its complications have also been well studied. Some compounds that specifically inhibit iron death have not yet entered clinical stages. However, studies have shown that some drugs in the market inhibit ferroptosis and are beneficial for diabetes and its complications. Rosiglitazone is the strongest inhibitor of ACSL4 (117) and ACSL4 is an essential component of ferroptosis (118). Rosiglitazone ameliorates DKD by maintaining kidney function and inhibiting the production of pro-inflammatory cytokines by inhibiting ACSL4 and blocking renal tubular cell ferroptosis (23). Fenofibrate is a third-generation fibric acid derivative widely used to treat patients with atherogenic dyslipidemia (119). Many studies have shown that fenofibrate can improve diabetes complications, but this effect does not depend on its ability to improve lipid levels (120). Fenofibrate delays the progression of DKD by inhibiting diabetes-related ferroptosis *via* upregulation of Nrf2 (120). Metformin is a commonly used anti-diabetic agent (121). Metformin attenuates hyperlipidemia/PA-induced vascular calcification through anti-ferroptosis effects (122). It is possible that metformin may also exert its anti-diabetic effects through this pathway, but further studies are needed to confirm this hypothesis.

Some compounds from natural products have been developed to protect cells against ferroptosis. Quercetin is one of the most widely distributed natural polyphenolic flavonoids in the plant kingdom (123). It has been reported to have potential anti-diabetic effects both *in vivo* and *in vitro* (124, 125). Epidemiological investigations have demonstrated that quercetin can reduce the risk of T2DM (124–127). Li et al. (60) investigated the mechanisms by which quercetin protects against T2DM and found that quercetin exerts beneficial effects on T2DM by inhibiting pancreatic iron deposition and pancreatic beta cell ferroptosis. Resveratrol, a natural polyphenol, inhibits acrolein-induced ferroptosis in MIN6 cells (77). Germacrone, one of the main bioactive components extracted from *Curcuma zedoaria Roscoe*, has been reported to have numerous pharmacological activities, including anti-inflammatory, anti-tumor, and anti-fibrotic effects (128, 129). Jin et al. found that germacrone combined with exogenous mmu_circRNA_0000309 facilitated DKD treatment by inactivating ferroptosis-dependent mitochondrial injury and podocyte apoptosis (130). Cryptochlorogenic acid was the main active component in the mulberry leaf extract. The extracts of mulberry leaf have been confirmed to improve inflammation and insulin resistance (131). Cryptochlorogenic acid exerts excellent anti-diabetic effects by inhibiting ferroptosis by activating cystine/xCT/Gpx4/Nrf2 and inhibiting NCOA4 in diabetes (132). These natural products have potential anti-ferroptosis properties; however, the targets of these compounds need to be further verified. Furthermore, randomized clinical trials of these anti-ferroptotic compounds for diabetic complications need to be conducted.

## Conclusions and Perspectives

**Figure 3:**
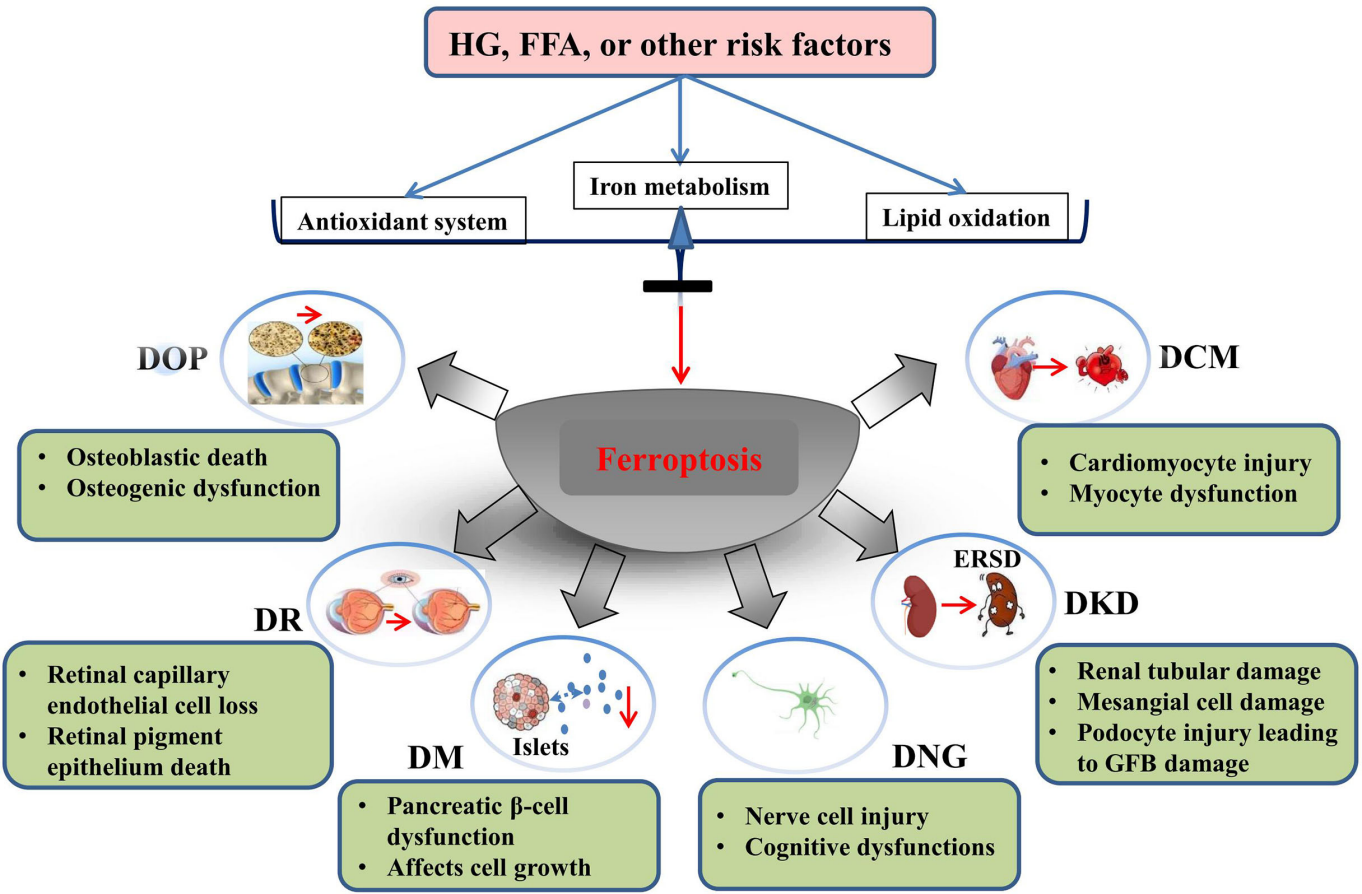
Ferroptosis is involved in the development of diabetes and related complications. HG and other risk factors (FFA or H/R) could induce ferroptosis by interfering with the balance of ion metabolism, reduction system, and lipid oxidation. Ferroptosis is involved in cell death of target organ and dysfunction in diabetes and its metabolic diseases.

In this review, we summarize the role and possible mechanism of ferroptosis in diabetes and its complications. To date, studies have demonstrated that ferroptosis plays an important role in diabetes and its complications, and drugs or agents that target ferroptosis may provide new treatment strategies for patients with diabetes. Morphologically, ferroptotis-experiencing cells, including HK-2, MC3T3, and hFOB 1.19 cells, exhibited reduced mitochondrial volume and cristae, and increased mitochondrial membrane density in diabetes (**Table 1**). These changes are consistent with the mitochondrial phenotypic changes induced by erastin. Moreover, HG and other risk factors (FFA or H/R) could induce ferroptosis in target cells through the following three categories: iron metabolism, antioxidant system, and lipid oxidation pathway. These defects may predispose cells to ferroptosis in diabetes (**Figure 3**).

**Table 1:** The main regulatory mechanism, morphological and biochemical feature of ferroptosis in diabetes and related complications.

Type of disease	Cell line/animal/clinical samples	Mechanism	Morphological features	Biochemical features	Reference
DKD	Animal: STZ-induced diabetic mice and db/db mice Cell: NRK-52E cells and HK-2 cells	ACSL4 was mediated ferroptosis.	Ruptured mitochondrial membrane and disappeared mitochondrial cristae.	Increase in ACSL4, MDA and iron content. Decreased in Gpx4.	Wang Y, et al. (23)
	Cell: Renal mesangial SV40-MES 13 cells Clinical samples: blood samples collected from DKD patients or healthy subjects	HMGB1/Nrf2 regulates HG-induced ferroptosis.	N.A.	Increase in ROS, MDA, ACSL4 and LDH release Decreased Gpx4 expression.	Wu Y, et al. (24)
	Animal: db/db mice	HIF-1α/HO-1 pathway might be regulates ferroptosis.	N.A.	Decrease in Gpx4, GSH-Px, CAT, SOD. Increase in MDA, ROS. Elevated serum iron ion, ferritin and transferrin.	Feng XM, et al. (25)
	Cell: NRK-52E cells Animal: STZ-induced diabetic mice Clinical samples: human kidney sample	N.A.	Mitochondria shrinkage and vanishing of mitochondrial cristae.	Decrease in SLC7A11 and Gpx4. Decrease in *xCT* and GSH. Increase in MDA, 4-HNE and FTH1.	Kim S, et al. (26)
	Animal: STZ-induced DBA/2J diabetic mice Cell: NRK-52E cells and HK-2 cells	Nrf2 inhibits ferroptosis.	Shrunken mitochondria with increased membrane density and ridge reduction or even disappearance.	Iron content, 4-HNE, MDA overload. Decrease in Gpx4, SLC7A11, and FTH-1. Increase in TFR-1.	Li SW, et al. (27)
	Cell: HK-2 cells	Salusin−β/Nrf−2 participates in HG−induced HK−2 cell ferroptosis.	N.A.	Decrease in Gpx4, GSH, SLC7A11, and FTH-1. Increase in TFR-1, iron content, MDA, ROS, and LDH release.	Wang WJ, et al. (88)
	Cell: MPC5 cells	Prdx6 negatively regulates HG-induced ferroptosis	N.A.	ROS, MDA, and iron overload. Decrease in SOD, GSH, and SLC7A11.	Zhang QJ, et al. (92)
DCM	Animal: DM+I/R rats Cell: H9c2 cells	ERS aggravates H/R or I/R-induced ferroptosis in cardiomyocytes.	N.A.	Increase in ACSL4, ATF4. Increase in LDH release, MDA, ROS and Fe^2+^. Decrease in SOD and Gpx4.	Li WY, et al. (29)
	Animal: Hsf1^+/+^ and Hsf1^-/-^ mice Cell: H9c2 cells	HSF1 regulates PA-induced ferroptosis in cardiomyocytes.	N.A.	Fe^2+^, MDA, and ROS overload. Decrease in Gpx4, FTH1 and SLC40A1.	Wang N, et al. (30)
	Animal: Nrf2 transgenic mice or Nrf2 KO mice treated with STZ. Cell: H9c2 cells	Nrf2 mediated ferroptosis in DCM.	N.A.	Iron deposition Increase in 4-HNE and ACSL4. Decrease in Gpx4 and Fsp1.	Zang HM, et al. (100)
DR	Cell: Human RCEC cells	TRIM46 regulates Gpx4 by promoting Gpx4 ubiquitination and degradation.	N.A.	Increase in MDA and lipid ROS. Decrease in SOD, Gpx4 and GSH-Px	Zhang JF, et al. (63)
	Cell: ARPE19	Circ-PSEN1 regulates HG-induced ferroptosis *via* miR-200b-3p/cofilin-2 axis.	N.A.	Fe^2+^ and MDA overload. Decrease in GSH, Gpx4 and SLC7A11. Increase in TRF1.	Zhu ZL, et al. (64)
Diabetic cognitive disease	Animal: STZ-induced diabetic rats	N.A.	Area of mitochondria was decreased. The sputum was shorter and diminished.	Fe^2+^ and 4-HNE overload. Decrease in SLC40A1.	Hao LJ, et al. (31)
	Animal: db/db mice	N.A.	Mitochondria were reduced and shrunken.	Fe content overload. Inactivation of SOD and GSH-Px. Increase in ACSL4 and TFR1. Decrease in FTH, FPN1, GPX4, FtMt, and SLC7A11.	An, JR, et al (105).
DOP	Cell: MC3T3 cells Animal: a rat model of DOP using intralipids and low-dose STZ	N.A.	Mitochondria appeared smaller and less tubular, membrane with distinct disrupted inner membrane folding	Accumulation of MDA and ROS. Decrease in GSH, SOD, Gpx4, Nrf2, and SLC7A11.	Ma HD, et al. (20)
	Cell: hFOB 1.19 cells Animal: a rat model of diabetic osteoporosis using high-fat and low-dose STZ	Mitophagy is mediated FtMt deficiency- promoted ferroptosis.	Mitochondria appeared smaller.	Increase in FtMt and DMT1. Decrease in Gpx4, Nrf2, and SLC7A11. ROS and lipid peroxidation overload.	Wang XD, et al. (67)
	Cell: HGPA (high glucose and palmitic acid)-treated MC3T3 cells. Animal: a rat model of DOP using HGHF (high glucose and high fat) and low-dose STZ.	METTL3/ASK1-p38 pathway regulates ferroptosis.	Mitochondria appearing shrunken, decreased cristae, and ruptured membrane.	Decrease in Gpx4 and SLC7A11.	Lin YF, et al. (114)

Further studies are needed to understand the regulatory mechanisms of ferroptosis in diabetes and its related complications. At present, there is a lack of knowledge regarding potential biomarkers that can be used to specifically diagnose ferroptosis in clinical settings. Although proteins related to iron metabolism, such as ferritin, are elevated in the serum of patients with diabetes and related complications, their specificity cannot meet the requirements of clinical diagnosis. Therefore, identification of biomarkers for ferroptosis will benefit the treatment of diabetes and its complications. Techniques for detecting ferroptosis *in vivo* should be developed in the future. In addition, the identification and development of specific inhibitors for ferroptosis is crucial, and much work remains to be done before specific inhibitors can be used clinically. In some cases, at least two or more types of programmed cell death may occur because of diabetic complications. Therefore, targeting of apoptosis, autophagy, necrosis, or ferroptosis alone may not achieve the desired effects, and multi-target therapeutics may be effective for treating complex diseases in the future.

## Author Contributions

X-DY wrote the manuscript. Y-YY contributions to design of the work and revised the work. All authors contributed to the article and approved the submitted version.

## Funding

This study was supported by the National Natural Science Foundation (No. 81603171), Hunan Provincial Natural Scientific Foundation (No. 2018JJ3743), and Hunan Traditional Chinese Medicine Science and Technology Project (No. 2021061).

## Conflict of Interest

The authors declare that the research was conducted in the absence of any commercial or financial relationships that could be construed as a potential conflict of interest.

## Publisher’s Note

All claims expressed in this article are solely those of the authors and do not necessarily represent those of their affiliated organizations, or those of the publisher, the editors and the reviewers. Any product that may be evaluated in this article, or claim that may be made by its manufacturer, is not guaranteed or endorsed by the publisher.
